# SAL0114: a novel deuterated dextromethorphan-bupropion combination with improved antidepressant efficacy and safety profile

**DOI:** 10.3389/fphar.2024.1464564

**Published:** 2024-09-24

**Authors:** Ying Xiao, Xuefeng Hu, Wei Xing, Jie Yan, Ruhuan Wang, Xiaoqing Li, Jiahuan Li, Zhixin Zhang, Jingchao Sun, Junjun Wu

**Affiliations:** The Guangdong Provincial Key Laboratory of Cardiovascular Drug R and D Enterprises, Shenzhen Salubris Pharmaceuticals Co., Ltd., Shenzhen, Guangdong Province, China

**Keywords:** deuterated dextromethorphan, bupropion, SAL0114, antidepressant efficacy, metabolic stability, C57BL/6J mouse, ICR mouse, SD rat

## Abstract

**Background:**

Esketamine, the first Food and Drug Administration-approved fast-acting antidepressant, has limited use because of its addictive properties. Although the combination of dextromethorphan and bupropion partially addresses the limitations of esketamine, concerns remain regarding neurologic side effects related to dextromethorphan metabolites, and seizure risks associated with high-dose bupropion. SAL0114, a novel formulation combining deuterated dextromethorphan (in which hydrogen atoms are replaced with deuterium) with bupropion, seeks to enhance dextromethorphan stability through deuteration of its metabolic sites. This approach is expected to increase antidepressant efficacy, reduce metabolite-induced safety issues, and allow for lower bupropion dosages.

**Methods:**

Radioligand competition binding assays were used to evaluate the impact of deuterium substitution on the *in vitro* activity of dextromethorphan and its metabolite, dextrorphan. *In vitro* hepatic microsomal stability and *in vivo* mouse pharmacokinetic assays were performed to assess the effects of deuteration on dextromethorphan stability. Two mouse models of behavioral despair were used to determine the antidepressant and synergistic effects of deuterated dextromethorphan and bupropion. Additionally, a reserpine-induced hypothermia rat model and an ammonia-induced cough mouse model were used to assess the *in vivo* effects from a pathological perspective.

**Results:**

Deuterated dextromethorphan maintained the same *in vitro* activity as dextromethorphan while exhibiting twice the metabolic stability both *in vitro* and *in vivo*. Combination with bupropion further improved its *in vivo* stability, increasing the exposure by 2.4 times. The combination demonstrated efficacy and synergistic effects in all tested animal models, showing superior efficacy compared with the dextromethorphan-bupropion combination.

**Conclusion:**

Deuteration improved dextromethorphan metabolic stability without altering its *in vitro* activity. Bupropion enhanced this stability and synergistically boosted the antidepressant effect by increasing deuterated dextromethorphan exposure *in vivo*. This enhanced metabolic stability suggests a reduction in dextromethorphan metabolites associated with clinical neurological side effects. Consequently, SAL0114 is hypothesized to offer improved efficacy and safety compared with the non-deuterated combination, potentially allowing for lower bupropion dosages. Further clinical studies are required to confirm these preclinical findings.

## 1 Introduction

Major depressive disorder (MDD) is a severe mental illness that affects millions of people worldwide (Li et al., 2021). Currently, there are five main classes of antidepressants: aminoketones, triazolopyridines, monoamine oxidase inhibitors, tricyclic antidepressants, and selective serotonin reuptake inhibitors. However, a common problem with these drugs is that they are slow to start working, often take weeks to take effect, and more than 30% of patients do not improve after treatment, putting them at risk of self-harm (Al-harbi, 2012).

Fortunately, the 2019 FDA-approved esketamine addresses the slow onset of depression by providing rapid relief within 24 h (FDA approves new nasal spray medication for treatment-resistant depression, available only at a certified doctor’s office or clinic, 2019). However, esketamine is highly addictive and currently limited in use in healthcare settings, which makes long-term medication management difficult for patients. Therefore, there is an urgent clinical need for safer and faster-acting antidepressants.

Dextromethorphan (DM) is an over-the-counter antitussive drug, with a mechanism of action similar to that of esketamine, both of which block NMDA receptors (Nguyen et al., 2014; Nguyen et al., 2017). Moreover, DM has been used as an antitussive agent for more than 40 years; therefore, it has a better safety potential than esketamine.

However, several issues must be addressed before administering dextromethorphan to patients with depression. Dextromethorphan has a strong hepatic first-pass effect that reduces its bioavailability and ultimately leads to diminished antidepressant effects (Pope et al., 2004; Taylor et al., 2016). In addition, dextromethorphan still has certain neurological side effects, including a potential risk of addiction, which are highly correlated with the metabolites of dextromethorphan (Clinical pharmacology and biopharmaceutics review for DextromethorphanDM+QuinidineQ, 2006; Schoedel et al., 2014). Therefore, metabolic enzyme inhibitors (CYP2D6 inhibitors) should be added to dextromethorphan to improve its stability. The FDA has approved combination medications, such as AVP-923 (DM + quinidine) and AXS-05 (DM + bupropion), based on similar tactics (Schoedel et al., 2014; Vecera et al., 2023). However, quinidine does not contribute to the antidepressant effect and has a cardiovascular safety risk that limits its dose, whereas bupropion has a risk of inducing epilepsy at high doses. Bupropion was withdrawn from the market because of this risk and was subsequently reintroduced to the market through formulation optimization, but this risk still exists. Therefore, in addition to the above strategy, we adopted a new strategy to deuterate the metabolic site of dextromethorphan. The aim of this strategy was to further improve the stability of dextromethorphan and reduce the dose of the CYP2D6 inhibitors.

By adopting these two strategies, we screened different combinations of compounds and selected the deuterated dextromethorphan (deDM)+ bupropion (BUP) combination. In this study, we evaluated the effect of deuterium substitution on the *in vitro* activity and *in vitro* and *in vivo* metabolic stability of deDM, in addition to the antidepressant effects of the deDM and BUP combination, and the comparative differences with the DM and BUP combination. These findings offer preclinical insights into the mechanism of action, synergistic effects, and potential safety of the combination of deDM and BUP in antidepressant treatment. Furthermore, they provide essential preclinical data to support and guide future clinical trials to ultimately benefit patients with depression.

## 2 Experimental materials and methods

### 2.1 Animals

Male Sprague–Dawley rats weighing 200–230 g and male C57BL/6J mice weighing 19–22 g were purchased from Zhejiang Vital River Laboratory Animal Technology Co., Ltd. (Zhejiang, China) and male ICR mice weighing 18–22 g were obtained from Shanghai Lingchang Biology Science and Technology Co., Ltd. (Shanghai, China). All rats and mice were housed in cages with 4–5 per cage under a 12 h light/dark cycle (8:00 a.m./8:00 p.m.) at a controlled temperature (25 °C ± 1°C) and humidity (55%) with free access to food and water. The animals were allowed to acclimatize for a week before the experiment. All procedures were performed in accordance with guidelines approved by the Institutional Animal Care and Use Committee of Shenzhen Salubris Pharmaceuticals Co., Ltd. (No.xlt-2021003, 2021.05.12; No. xlt-2021005, 2021.05.18) (Shenzhen, China), and Wuxi Apptec Co., Ltd. (GP01-054-2020v1.0) (Shanghai, China). Efforts were made to minimize animal suffering and counts.

### 2.2 Reagents and compounds

Deuterated dextromethorphan (deDM), deuterated dextrorphan (deDX), bupropion (BUP), dextromethorphan (DM), and dextrorphan (DX) were obtained from Shenzhen Salubris Pharmaceuticals Co., Ltd. (Shenzhen, China). Reserpine (catalog number: 83580) and imipramine (catalog number: D3900) were obtained from Sigma‒Aldrich (Shanghai, China); ammonium hydroxide (catalog number: A291784) was purchased from Ailan (Shanghai) Chemical Technology Co., Ltd. (Shanghai, China); and ^3^H-MK801 (catalog number: NET972250UC), ^3^H-hydroxytryptamine creatine sulfate (catalog number: NET498001MC), ^3^H-norepinephrine hydrochloride (catalog number: NET048250UC), ^3^H-imipramine (catalog number: NET576250UC), ^3^H-nisoxetine (catalog number: NET1084250UC), serotonin transporter membrane (catalog number: RBHSTM400UA) and norepinephrine transporter (catalog number: RBHNETM400UA) were purchased from PerkinElmer, Inc (Massachusetts, United States); NMDA membranes were obtained from Pharmron (Beijing, China); and memantine hydrochloride (catalog number: M9292) and paroxetine (catalog number: A11904-25) were purchased from Adooq Bioscience (California, United States); human liver microsomes (catalog number: LM-R-02M) were purchased from Research Institute for Liver Diseases Co. Ltd. (Shanghai, China); BCA protein assay kit was purchased from Thermo Fisher Scientific Inc. (catalog number: 23227, Massachusetts, United States).

### 2.3 Radioligand competition binding

#### 2.3.1 NMDAR binding

Membrane preparation was performed as previously described (Werling et al., 2007). Briefly, the brains of male Sprague-Dawley rats were dissected for tissue collection. The tissue was then homogenized in ice-cold extraction solution and centrifuged at 40,000 × g for 10 min (minute). The supernatant was discarded, the precipitate was resuspended in extraction solution, and the above steps were repeated two times. Finally, the precipitate was resuspended in a 10-fold volume of resuspension solution and stored at −80°C. Protein concentration was determined using a PierceTM BCA Protein Assay Kit. The competitive binding of the compound and ^3^H-MK801 to the NMDA receptor was evaluated utilizing the filtration binding method. This method involves washing unbound radioligands and subsequently quantifying the signal values of the radioligands bound to the NMDA receptor using a Microbeta instrument (catalog number: 2450, Perkin Elmer, Massachusetts, United States).

#### 2.3.2 Serotonin transporter (SERT) binding

The experiments were carried out in the Pharmaron laboratory, and the methods were referenced from previous studies with modifications (Yang and Gouaux, 2021). Competitive binding of the samples and ^3^H-hydroxytryptamine creatine sulfate on SERT was assessed using a radioligand binding method. The ability of the compound to competitively bind to SERT (Ki) was determined by detecting the signal value of the radioligand bound to the transporter using a Microbeta instrument (2,450, Perkin Elmer, Massachusetts, United States).

#### 2.3.3 Norepinephrine transporter (NET) binding

The experiments were carried out in the Pharmaron laboratory, and the methods were referenced from previous studies with modifications (Bylund and Toews, 1993; Galli et al., 1995). Competitive binding of the compound and ^3^H-Nisoxetine to NETs was assessed using a radioligand binding method. The ability of the compound to competitively bind to NET (Ki) was determined by detecting the signal value of the radioligand bound to the transporter using a Microbeta instrument (2450, Perkin Elmer, Massachusetts, United States).

#### 2.3.4 Sigma 1 receptor binding

The experiments were carried out in the Eurofins Discovery laboratory, and the methods were referenced from previous studies with modifications (Prasad et al., 1999). Competitive binding of the samples and ^3^H-pentazocine to the Sigma-1 receptor (Sigma-1R) of human-derived Jurkat cells was assessed using a radioligand binding method. The ability of the compound to bind competitively to the Sigma-1 receptor (Ki) was determined by detecting the signal value of the radioligand bound to the receptor.

#### 2.3.5 Nicotinic acetylcholine receptor (nAchR) α3β4 binding

The experiments were carried out in the Eurofins Discovery laboratory, and the methods were referenced from previous studies with modifications (Michelmore et al., 2002; Zaveri et al., 2010). The competitive binding of the samples and ^125^I-epibatidine to the nAchR α3β4 in human recombinant CHO-K1 cells was assessed using a radioligand binding method. The ability of the compound to competitively bind to nAchR α3β4 (Ki) was determined by detecting the signal value of the radioligand bound to the receptor.

### 2.4 Metabolic stability in liver microsomes

The assay was built in a way similar to that previously published by Zhang et al. (Zhang et al., 2018) with some modifications. deDM or DM (1 μM) was incubated in 0.5 mg/mL human liver microsomes in 0.1 M phosphate buffer at 37°C. All incubations were performed in duplicates. Verapamil was used as the positive control. After pre-warming the mixture for 5 min, reactions were initiated by the addition of NADPH (1 mM). Samples (30 μL) were taken at 0, 20, and 60 min, and the reaction was immediately terminated by adding 150 μL acetonitrile containing an analytical internal standard (IS). Samples were centrifuged at 4,000 rpm for 5 min in a centrifuge (5810R, Eppendorf, Germany), and the supernatant was analyzed by LC-MS/MS (Thermo Scientific TSQ Quantum Ultra, United States).

CL_int, *in vitro*_ (the *in vitro* intrinsic clearance, CL_int, *in vitro*_= (0.693/t1/2) × (1/C_protein_)) was calculated from the t_1/2_ of deDM or DM disappearance, where C_protein_ is the liver microsomes protein concentration during the incubation, and t1/2 was determined by the slope (k) of the log-linear regression analysis of the concentration versus time profiles; thus, t_1/2_ = ln2/k. The *in vivo* clearance values (CL_int_ = CL_int, *in vitro*_× (mg protein/g liver weight) × (g liver weight/kg body weight)) of humans were predicted by CL_int, *in vitro*_ values using physiologically-based scaling factors, hepatic microsomal protein concentrations (48.8 mg protein/g liver), and liver weights (25.7 g/kg body weight). The *in vivo* hepatic clearance (CL_H_= (Q × CL_int_)/(Q + CL_int_)) was then calculated by using CL_int_ and hepatic blood flow, Q (20.7 mL·min^−1^·kg^−1^ in humans).

### 2.5 Pharmacokinetic study in C57BL/6 mice

The assay was built in a way similar to that previously published by Beconi et al. (2011) with some modifications. A total of 36 male C57BL/6 mice, weighing between 20 and 22 g, were randomly divided into four groups of nine mice each. The four groups were as follows: DM (20 mg/kg), deDM (20 mg/kg), DM + BUP (20 mg/kg + 120 mg/kg), and deDM + BUP (20 mg/kg+120 mg/kg). Mice in each group were numbered 1–9. Following the oral administration of the compounds in each group, blood was collected from the jugular vein at the specified time points, with three mice at each time point and two to three times for each mouse. 1–3 numbered mice had blood collection time points of 0.25, 1.5, and 7 h after the administration of the compounds, 4–6 numbered mice had blood collection time points of 0.5, 2, and 24 h after the administration of the compounds, and 7–9 numbered mice had blood collection time points of 1 and 5 h after the administration of the compounds. All blood samples were collected without anesthesia, and blood was collected in heparinized EP tubes. Plasma was obtained by centrifuging the blood at 10,000 rpm for 2 min at 4°C. The concentrations of deDM and DM in mouse plasma were determined using an LC-MS/MS assay (TSQ Quantum Ultra, Thermo Scientific, Massachusetts, United States). The mean deDM or DM concentrations per time point were used to calculate the composite PK parameters through non-compartmental analysis using WinNonlin 8.1.

### 2.6 Forced swim test

The mouse forced swim test was performed in a way similar to that published previously by Nguyen et al. (Nguyen et al., 2017) with some modifications. 60 male ICR naïve male ICR mice were randomly assigned to six groups (n = 10 per group): vehicle (saline), BUP (50 mg/kg), low-dose deuterated deDM (10 mg/kg), high-dose deDM (18 mg/kg), low-dose deDM + BUP (10 mg/kg + 50 mg/kg), and high-dose deDM + BUP (18 mg/kg + 50 mg/kg). All compounds were administered orally via gavage at a volume of 10 mL/kg body weight, with the vehicle group receiving an equivalent volume of saline. 1 h after administration, mice underwent the forced swim test (FST), a widely used behavioral assay for evaluating antidepressant-like effects. Each mouse was individually placed in a transparent cylindrical tank filled with water (depth: 18 cm, temperature: 25°C ± 1°C) for a 6-min test session. The initial 2 min served as the acclimatization period and was not scored. Floating time during the subsequent 4 min was measured using ANY-Maze Version 4.63 video tracking software (Stoelting Co., Illinois, United States). Floating was defined as the minimum movement required to keep the animal’s head above water. Floating time (%) = floatting time(s)/240 s. The ANY-Maze software parameters were set as follows: acclimatization period = 120 s, test duration = 240 s, minimum immobility time = 2 s, and immobility sensitivity = 75%. To maintain consistent conditions, water was changed between the animals. All experimental procedures were conducted in strict accordance with guidelines approved by the Institutional Animal Care and Use Committee of Wuxi Apptec Co., Ltd. A *P* value of less than 0.05 was considered statistically significant for comparisons between groups.

### 2.7 Tail suspension test

The TST was adapted from the behavioral despair test described by Nguyen et al. (Nguyen et al., 2017), with some modifications. 60 male naïve male ICR mice were randomly assigned to six groups (n = 10 per group): vehicle (saline), BUP (50 mg/kg), low-dose deuterated deDM (10 mg/kg), high-dose deDM (18 mg/kg), low-dose deDM + BUP (10 mg/kg + 50 mg/kg), and high-dose deDM + BUP (18 mg/kg + 50 mg/kg). All compounds were administered orally via gavage at a volume of 10 mL/kg body weight, with the vehicle group receiving an equivalent volume of saline. One hour after administration, mice underwent a tail suspension test (TST). For the TST, each mouse was suspended by its tail, with adhesive tape attached 2 cm from the tail tip and secured to a metal rod. Care was taken to ensure a minimum distance of 15 cm between the suspended animal and the surrounding objects. The test was performed using the ANY-maze version 4.63 video tracking program (Stoelting Co., Wood Dale, IL, United States) for a total duration of 6 min. The initial 2 min served as an acclimatization period and were not scored. The immobility time during the subsequent 4 min was measured using the same software. Immobility was defined as mouse hanging passively and motionless. The ANY-Maze software parameters were set as follows: acclimatization period = 120 s, test duration = 240 s, minimum immobility time = 2 s, and immobility sensitivity = 75%. All experimental procedures were conducted in strict accordance with the guidelines approved by the Institutional Animal Care and Use Committee of Wuxi Apptec Co., Ltd. A *P* value of less than 0.05 was considered statistically significant for comparisons between groups.

### 2.8 Reserpine-induced hypothermia in rats

A reserpine-induced hypothermia test was performed according to the method described by Rojas-Corrales et al. (Rojas-Corrales et al., 2004; Dhamija et al., 2017), with some modifications. 80 male Sprague-Dawley rats were administered an intraperitoneal injection of reserpine (4 mg/kg). 18 h after the injection, rectal temperatures were measured using a rectal probe connected to a thermometer (MC-347, Omron Healthcare, Dalian, China). Rats with body temperatures not exceeding 34.5°C were considered successfully modeled. 70 eligible rats were randomly divided into seven groups (n = 10 per group): vehicle (saline), BUP (40 mg/kg), low-dose deDM (5 mg/kg), high-dose deDM (10 mg/kg), low-dose deDM + BUP (5 mg/kg + 40 mg/kg), high-dose deDM + BUP (10 mg/kg + 40 mg/kg), and DM + BUP (5 mg/kg + 40 mg/kg). An additional group of ten rats not treated with reserpine served as the normal control. After the group assignment, the second phase of compound administration was initiated. The normal and model control groups received oral saline (10 mL/kg), whereas the other groups received their respective compounds orally at the specified doses, all in an equivalent volume (10 mL/kg). Rectal temperatures were measured 30 min after treatment to assess the effect of compounds in each group on the reserpine-induced hypothermia model. All experimental procedures were conducted in strict accordance with guidelines approved by the Institutional Animal Care and Use Committee of Shenzhen Salubris Pharmaceuticals Co., Ltd. A *P* value of less than 0.05 was considered statistically significant for comparisons between groups.

### 2.9 Ammonia-induced cough mouse model

Antitussive effects were investigated using a classical mouse cough model induced by ammonia liquor, with some modifications (Jiang et al., 2014; Adejayan et al., 2019). An ammonia-induced cough model in male C57BL/6J mice was used to evaluate the antitussive effects using a two-step approach. The initial screening phase aimed to identify the mice with consistent and pronounced cough responses. Individual mice were exposed to 0.2 mL of 13% ammonium hydroxide solution in 500 mL glass vials. A trained observer measured the latency period and recorded the number of coughs over a 3-min period post-exposure. Mice demonstrating more than three coughs within 1 minute and a latency period shorter than 1 minute were selected for the study, ensuring a cohort with robust cough responses. Following a 24-h recovery period, 70 eligible mice were randomly allocated to seven treatment groups (n = 10 per group) for the compound evaluation phase. The groups were as follows: vehicle (saline), BUP (40 mg/kg), low-dose deDM (5 mg/kg), high-dose deDM (10 mg/kg), low-dose deDM + BUP (5 mg/kg + 40 mg/kg), high-dose deDM + BUP (10 mg/kg + 40 mg/kg), and DM + BUP (5 mg/kg + 40 mg/kg) groups. All compounds were administered orally at a volume of 10 mL/kg. At 0.5 h post-administration, each mouse was re-exposed to 0.2 mL of 13% ammonia in a 500 mL glass jar for 1 min. Subsequently, the trained observer recorded the number of coughs over a 3-min period following removal from the jar. Antitussive efficacy was quantified using the cough inhibition rate, calculated as follows: Inhibition rate (%) = [(To-T)/To ×100%], Where To = number of coughs in the vehicle group and Tt = number of coughs in the treatment group. All experimental procedures were conducted in strict compliance with guidelines approved by the Institutional Animal Care and Use Committee of Shenzhen Salubris Pharmaceuticals Co., Ltd., ensuring adherence to ethical standards in animal research. A *P* value of less than 0.05 was considered statistically significant for comparisons between groups.

### 2.10 Statistical analysis

SPSS 16.0 was used for statistical analysis of data. The groups were first tested for normality, followed by a data homogeneity test using Levene’s test. Statistical analysis was performed to analyze significant differences between the groups. Non-parametric tests were used for non-normal groups. For groups with a normal distribution, one-way ANOVA was used. A *P* value of less than 0.05 was considered to indicate statistical significance. Data are presented as mean ± standard error of the mean (SEMs). The IC_50_ values were calculated using GraphPad Prism 5.0.

## 3 Results

### 3.1 Radioligand competition binding

We evaluated the competitive binding ability of the four compounds to five targets in relation to their antidepressant effects. The compounds tested included DM, DX (a metabolite of DM), deDM, and deDX (a metabolite of deDM). The specific competitive binding of the four compounds to these targets is shown in Table 1 and Supplementary Figures S1–S5. According to the data presented in Table 1, DM had excellent affinity for SERT (Ki = 0.0084 μM), followed by NMDA receptor (Ki = 1.59 μM) and sigma-1 receptor (Ki = 1.67 μM), and then nAch α3β4 receptor (Ki = 14.70 μM) and NET (Ki = 20.51 μM). Based on the experimental fluctuations in background values, Ki was considered to have a comparable affinity in the 2-fold range. deDM showed similar affinity profiles to those of DM for all five targets. On the other hand, DX showed good affinity for both NMDA receptor (Ki = 0.22 μM) and SERT (Ki = 0.14 μM), followed by sigma-1 receptor (Ki = 2.85 μM), NET (Ki = 5.67 μM), and nAch α3β4 receptor (Ki = 9.98 μM). deDX showed similar affinities to DX for all five targets. Notably, DX had a higher affinity for NMDA receptors (7-fold) and a lower affinity for SERT (17-fold) compared to DM.

**TABLE 1:** Radioligand binding data for dextromethorphan (DM), dextrorphan (DX), deuterated dextromethorphan (deDM) and deuterated dextrorphan (deDX).

Receptor or transporter	DM, Ki (μM)	deDM, Ki (μM)	DX, Ki (μM)	deDX, Ki (μM)
NMDA receptor	1.59	1.90	0.22	0.42
Sigma-1 receptor	1.67	1.72	2.85	2.86
α3β4 nicotinic acetylcholine receptors	14.70	15.80	9.98	15.0
serotonin transporter	0.0084	0.0083	0.14	0.12
norepinephrine transporter	20.51	17.08	5.67	7.93

Results expressed as mean, n = 4 for NMDA, receptor, n = 2 for other radioligands competitive binding assay.

### 3.2 *In Vitro* metabolism by liver microsomes

As shown in Table 2, in the *in vitro* assay of human hepatic microsomal stability, deDM exhibited a lower clearance rate, and consequently, a longer half-life compared to DM. The half-life of deDM was observed to be approximately twice that of DM.

**TABLE 2:** Metabolic stability of Compoud in human liver microsomes.

Compound	T_1/2_ (min)	CL_int,_ *_in vitro_* (mL/min/kg)	CL_H_ (mL/min/kg)
Dextromethorphan	74.2	23.4	11.0
Deuterated dextromethorphan	135.9	12.8	7.9

Results expressed as mean, n = 2.

### 3.3 Pharmacokinetic study in C57BL/6 mice

As shown in Table 3, the AUC_0-last_ and C_max_ values of deDM were higher than those of DM at the same dose. The exposure of deDM (AUC_0-last_) was approximately twice that of DM, suggesting that deuterium may enhance the stability of DM in mice. When deDM was combined with BUP, the AUC_0-last_ and C_max_ values of deDM were found to be further increased. This combination resulted in a 2.4-fold higher exposure than deDM alone, indicating that BUP may further enhance the stability of deDM in mice. Additionally, BUP also increased DM exposure. At the same dose, the AUC_0-last_ and C_max_ of deDM in the combination of deDM and BUP were higher than those of DM in the combination of DM and BUP, suggesting that deDM in the combination with BUP is more stable than DM in the combination with BUP in mice.

**TABLE 3:** Pharmacokinetic parameters of DM or deDM in C57BL/6 mice after oral administration of the following different groups of treatment.

Group	Dose (mg/kg)	AUC_0-last_ (h*ng/mL)	C_max_ (ng/mL)
DM	20	184 ± 168	74.9 ± 49
deDM	20	387 ± 163	269 ± 83
DM + BUP	20 + 120	652 ± 437	315 ± 157
deDM + BUP	20 + 120	940 ± 329	438 ± 123

Results expressed as mean ± SD, n = 9 per group.

### 3.4 Behavioral despair model

**FIGURE 1:**
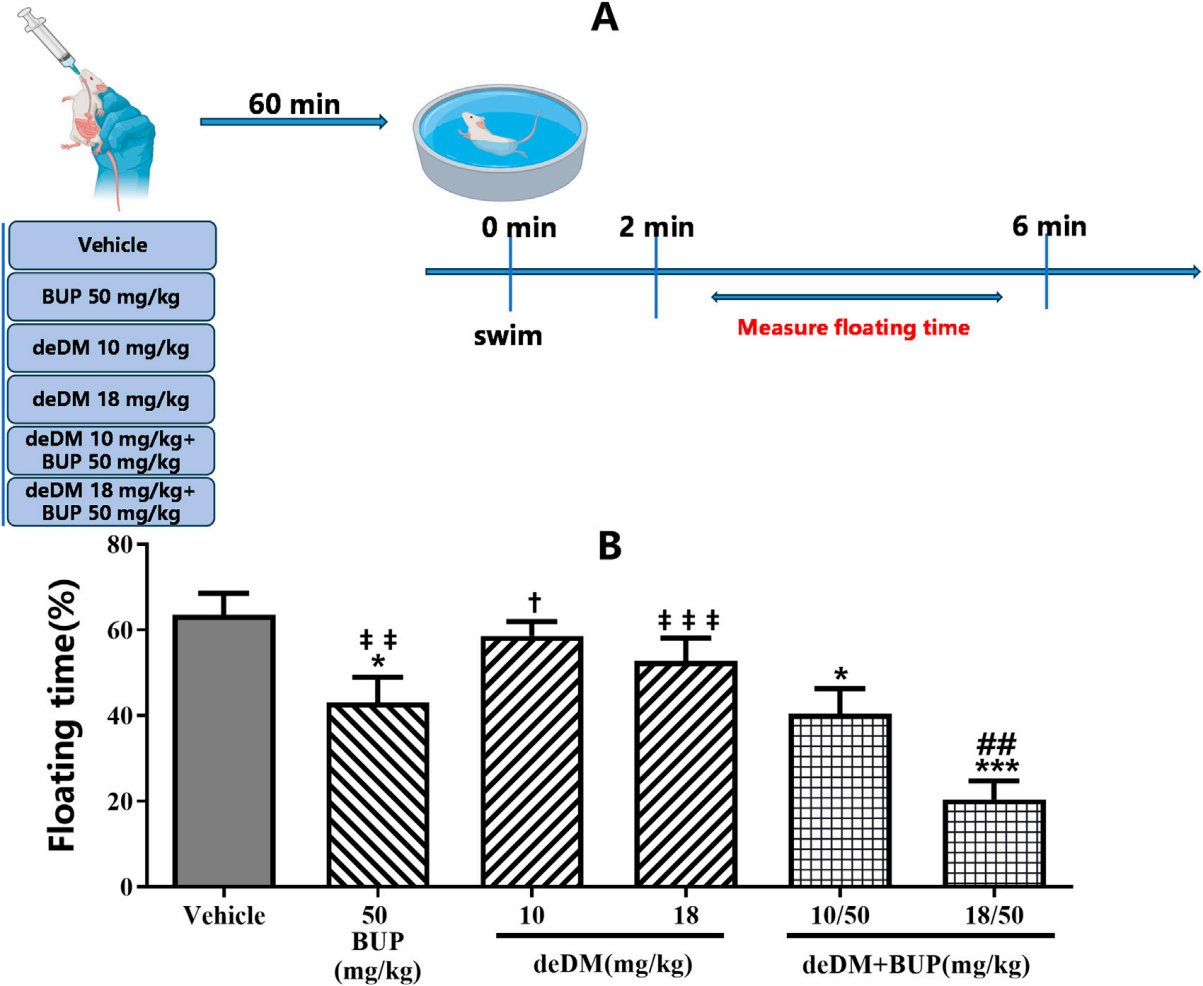
Antidepressant-like effects of compounds in the forced swim test in mice **(A)** Experimental design overview. **(B)** Floating time (%). The data shown are expressed as the mean ± S.E.M. *P < 0.05, ***P < 0.001, compared with the saline-treated group, ##*P* < 0.01, compared with the BUP-treated group via one-way ANOVA followed by Dunnett’s test; †*P* < 0.05, deDM (10 mg/kg) and deDM + BUP (10 mg/kg + 50 mg/kg) were compared, ‡‡*P* < 0.01, ‡‡‡*P* < 0.001, deDM (14 mg/kg) and BUP (50 mg/kg) were compared with the combination of deDM + BUP (14 mg/kg + 50 mg/kg) via one-way ANOVA followed by SNK test; n = 10 per group; Vehicle represents the saline control group, BUP represents the bupropion group, deDM represents the deuterated dextromethorphan group, and deDM + BUP represents the deuterated dextromethorphan and bupropion combination group.

As shown in Figure 1, in the FST, deDM did not significantly reduce the floating time of mice compared to the vehicle group, but BUP significantly reduced the floating time of mice. When deDM was combined with BUP, the floating time of mice was significantly reduced in a dose-dependent manner. The low dose of deDM + BUP (10 mg/kg+50 mg/kg) was not significantly different from BUP and was significantly different from deDM (10 mg/kg); however, this significant difference was mainly contributed to BUP. Meanwhile, the high dose of deDM + BUP (18 mg/kg+50 mg/kg) not only had a significant difference with deDM but also had a significant difference with the BUP group, and it was extremely significant, which also reflected the synergistic effect of BUP and deDM.

**FIGURE 2:**
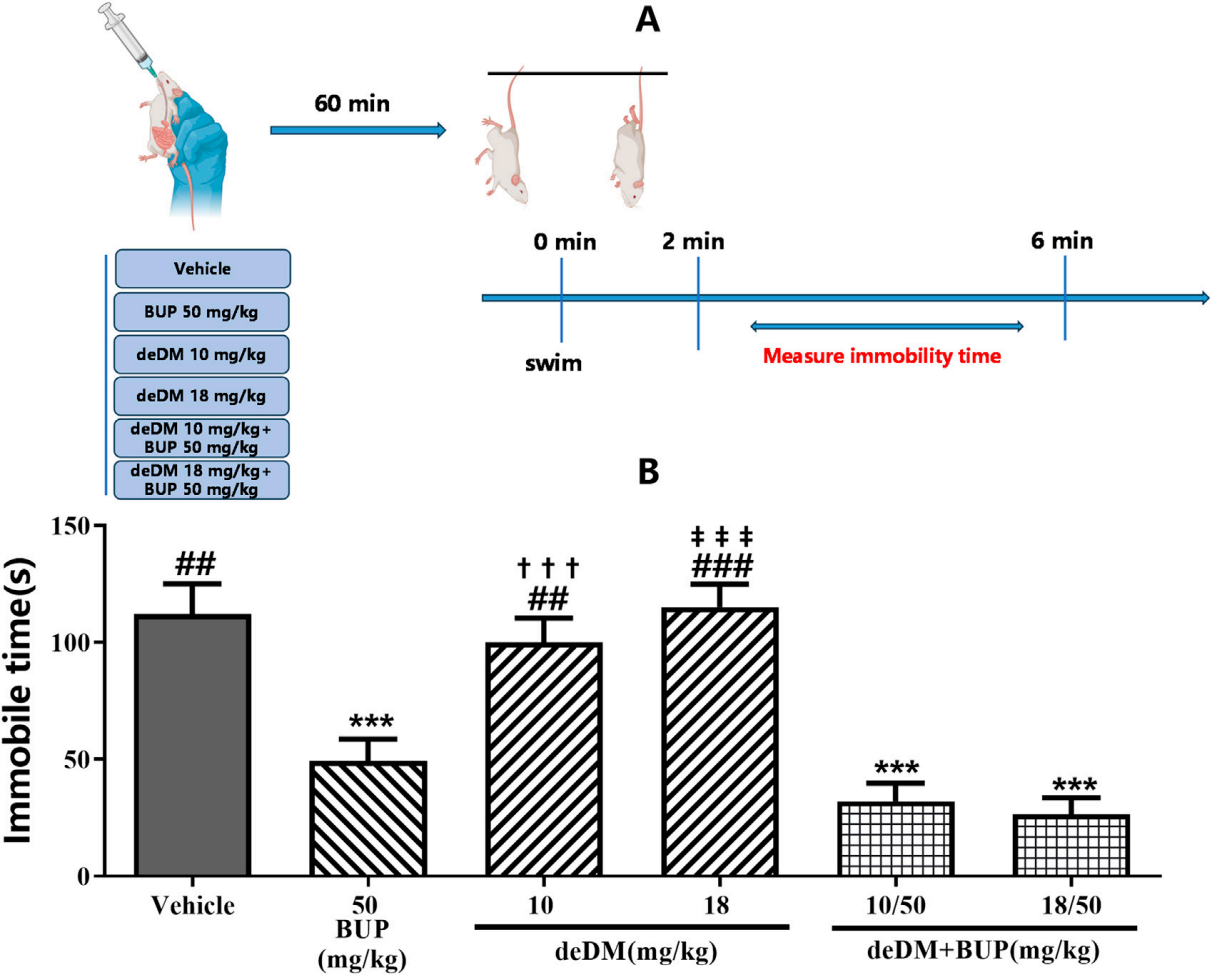
Antidepressant-like effects of compounds in the tail suspension test in mice **(A)** Experimental design overview. **(B)** Immobile time (s). The data shown are expressed as the mean ± S.E.M. ***P < 0.001, compared with the saline-treated group via one-way ANOVA followed by Dunnett’s test; †††*P* < 0.001, deDM (10 mg/kg) and deDM + BUP (10 mg/kg + 50 mg/kg) were compared, ‡‡‡*P* < 0.001, deDM (18 mg/kg) and deDM + BUP (18 mg/kg + 50 mg/kg) were compared via one-way ANOVA followed by SNK test; n = 10 per group; Vehicle represents the saline control group, BUP represents the bupropion group, deDM represents the deuterated dextromethorphan group, and deDM + BUP represents the deuterated dextromethorphan and bupropion combination group.

As shown in Figure 2, similar to the results of the FST, deDM did not significantly reduce the immobilization time of mice compared to the vehicle group, but BUP significantly reduced the immobilization time of mice. When deDM was combined with BUP, the immobilization time of mice was significantly reduced in a dose-dependent manner. However, no significant difference was observed between the two doses of deDM + BUP and BUP. Although there was a significant difference between the two doses of deDM + BUP compared with their respective single deDM, this difference was mainly attributed to BUP. The non-significant difference between the two doses of the deDM + BUP combination group and BUP may be related to the fact that the maximum plateau of efficacy was reached with the combination of deDM and BUP.

### 3.5 Rat reserpine-induced hypothermia model

**FIGURE 3:**
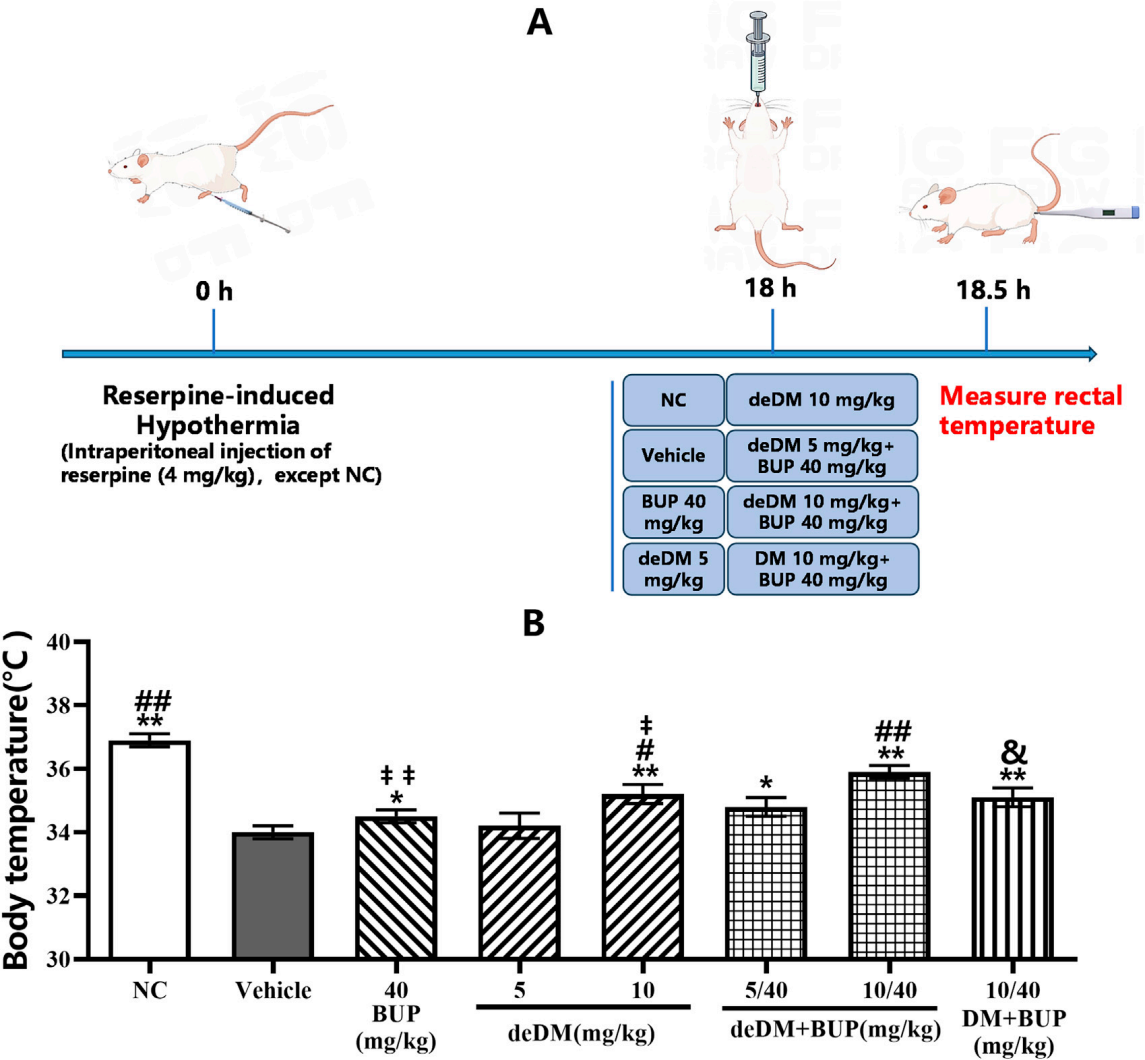
The effects of the compounds on reversing hypothermia in a rat reserpine-induced hypothermia model **(A)** Experimental design overview. **(B)** Body temperature (°C). The data shown are expressed as the mean ± S.E.M. *P < 0.05, **P < 0.01, compared with the saline-treated model control group, ##*P* < 0.05, ##*P* < 0.01, compared with the BUP-treated group via one-way ANOVA followed by Dunnett’s test; †*P* < 0.05, ‡‡*P* < 0.01, deDM (10 mg/kg) and BUP (40 mg/kg) were compared with the combination of deDM + BUP (10 mg/kg + 40 mg/kg) via one-way ANOVA followed by SNK test; &*P* < 0.01, deDM + BUP (10 mg/kg + 40 mg/kg) and DM + BUP (10 mg/kg + 40 mg/kg) were compared via one-way ANOVA followed by LSD test; n = 10 per group; NC represents the saline normal control group, Vehicle represents the saline model control group, BUP represents the bupropion group, deDM represents the deuterated dextromethorphan group, deDM + BUP represents the deuterated dextromethorphan and bupropion combination group; DM + BUP represents the dextromethorphan and bupropion combination group.

As shown in Figure 3, the body temperature of the rats in the model group was significantly lower than that of the normal control (NC) group, and bupropion significantly increased the body temperature of the rats compared with that of the model (vehicle) group, indicating that the hypothermia model was successfully established. deDM increased the body temperature of model animals in a dose-dependent manner, with a high dose of deDM (10 mg/kg) reaching a statistically significant difference. When deDM was combined with bupropion, the combination further increased the body temperature of rats in a dose-dependent manner. The low-dose combination of deDM + BUP (5 mg/kg + 40 mg/kg) did not show a significant difference in any of the groups compared with BUP and low-dose deDM, although there was a tendency for elevation. The high-dose deDM + BUP (10 mg/kg + 40 mg/kg) combination showed significant differences compared to the BUP and high-dose deDM groups, suggesting that BUP and deDM have some synergistic effects. In addition, the combination of deDM and BUP (10 + 40 mg/kg) was more potent than the combination of DM and BUP (10 + 40 mg/kg) at elevating the body temperature of rats at the same dose, and the difference between the two groups was significant. Although the different treatment groups increased the body temperature of the model rats to different degrees, none of them returned to the body temperature level of the NC group, which may be related to the fact that the model was an acute model and the dose design was low.

### 3.6 Ammonia-induced mouse cough model

**FIGURE 4:**
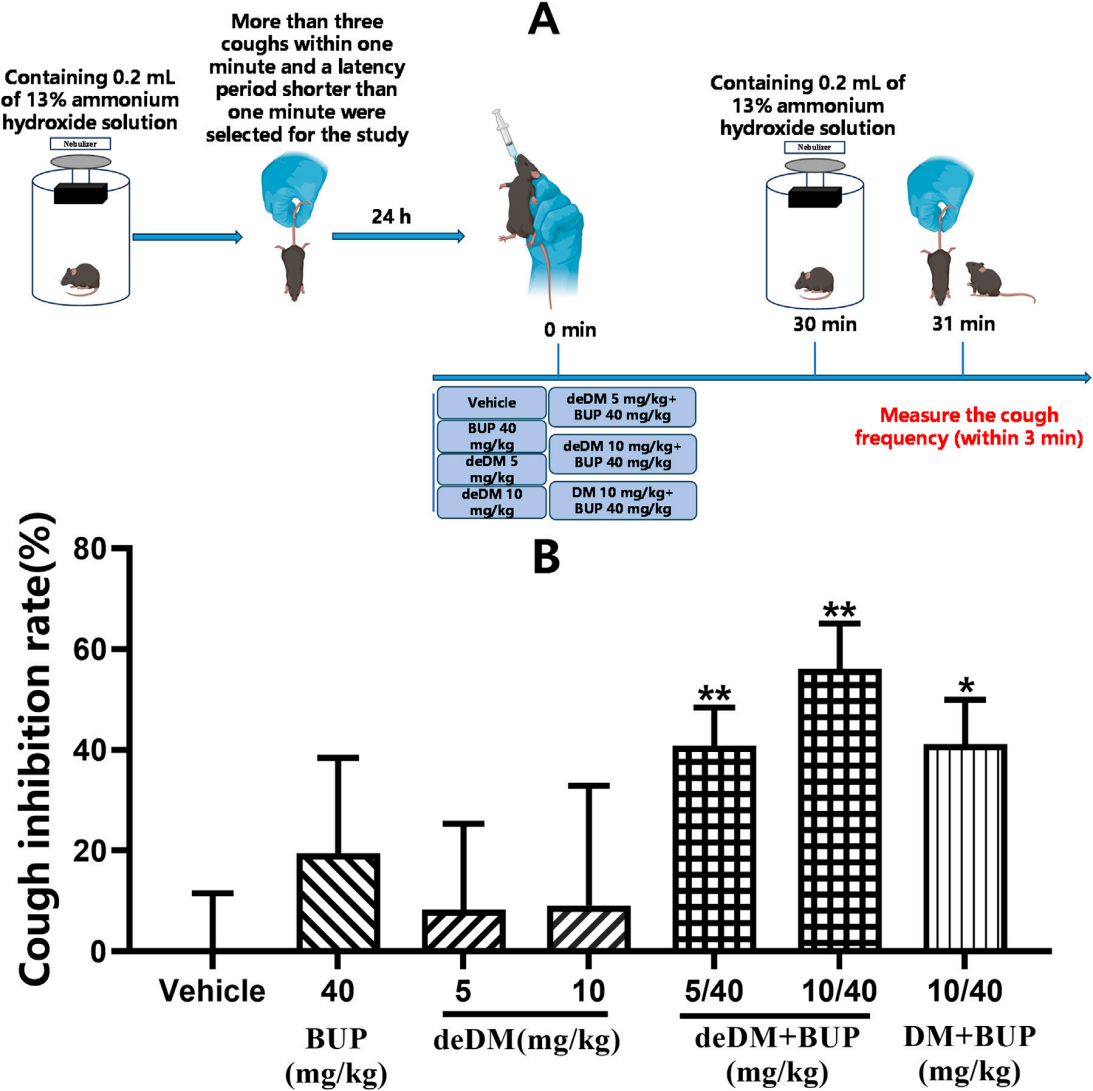
Antitussive effects of compounds in an ammonia-induced cough mouse model **(A)** Experimental design overview. **(B)** Cough inhibition rate (%). The data shown are expressed as the mean ± S.E.M. *P < 0.05, **P < 0.01, compared with the saline-treated group via one-way ANOVA followed by Dunnett’s test; n = 10 per group; BUP represents the bupropion group, deDM represents the deuterated dextromethorphan group, deDM + BUP represents the deuterated dextromethorphan and bupropion combination group, and DM + BUP represents the dextromethorphan and bupropion combination group.

As shown in Figure 4, neither deDM nor BUP significantly inhibited cough in mice compared with the vehicle group. However, when deDM and BUP were combined, the deDM + BUP combination inhibited cough in mice in a dose-dependent manner, with both the low and high doses of the deDM + BUP combination showing significant differences compared to the vehicle group. When comparing both doses of the deDM + BUP combination with their respective individual drugs, no significant differences were observed, which may be related to the higher variability in the effects of BUP and deDM alone. Notably, the inhibition of cough by the low-dose deDM + BUP combination (40.8%) was greater than the sum of cough inhibition by the low-dose deDM and BUP individual drugs (8.3% + 19.5%). Similarly, the high-dose combination also demonstrated greater efficacy than the sum of its individual components (56.1% > 9.1% + 19.5%). These data indirectly reflect the synergistic inhibition of coughing in mice with deDM and BUP. Additionally, the combination of deDM and BUP (10 + 40 mg/kg) exhibited a better inhibitory effect on cough than the combination of DM and BUP (10 + 40 mg/kg) at the same dose (56.1% vs. 41.1%, *p* > 0.05).

## 4 Discussion

In this study, we first investigated the effect of deuterium substitution on the activity of DM and DX via *in vitro* assays, which directly influences their antidepressant effects *in vivo*.

Previous studies (Stahl, 2019) have reported that DM can bind to multiple targets including NMDARs, sigma-1 receptors, nACh receptors, SERT, and NETs. We investigated the impact of deuteration on DM activity at these targets and found that deuteration did not affect DM activity. Similarly, we examined the activity of the DM metabolite DX and deuterated metabolite of deDM, deDX, and found that deuteration did not affect the activity of DX at these targets.

Furthermore, we investigated the effect of deuterium substitution on the metabolic stability of DM *in vitro* and *in vivo*. Our findings from *in vitro* hepatic microsomal stability assays and PK studies in mice indicate that deuterium substitution enhances the metabolic stability of DM *in vitro* and *in vivo* and increases compound exposure *in vivo*, which also implies that deuterium substitution decreases metabolite exposure. Research studies (Clinical pharmacology and biopharmaceutics review for DextromethorphanDM+QuinidineQ, 2006; Schoedel et al., 2014) have shown that the primary neurological adverse effects of DM include dizziness, somnolence, confusion, hallucinations, cognitive dysfunction, and dyskinesia. These adverse effects are significantly associated with DM metabolites and may primarily be attributed to the action of NMDA receptors. Our *in vitro* target competition binding assay showed that DX binds NMDAR more strongly than DM, with 7-fold activity, further suggesting that DX may cause more neurological adverse effects. Therefore, deuterium substitution may improve the metabolic stability of DM and reduce metabolite levels, which may lead to a better safety profile.

To further investigate the antidepressant efficacy of deDM and BUP *in vivo*, we used the FST and TST to evaluate the antidepressant effects of deDM, BUP, and their combination. FST and TST are classic models for screening antidepressant drugs, allowing for rapid assessment of antidepressant activity, and are widely used in drug screening and development (O’Leary and Cryan, 2009; Yankelevitch-Yahav et al., 2015). The results demonstrated that deDM had no significant effect on the floating or immobilization time of mice in the FST and TST at doses ranging from 10 mg/kg to 18 mg/kg. However, in combination with BUP, deDM significantly reduced the floating or immobilization time in mice in a dose-dependent manner. Furthermore, deDM + BUP (18 + 50 mg/kg) exhibited a more significant reduction in the floating time of mice in the FST than the BUP group. These findings indicated that deDM and BUP may act synergistically to produce antidepressant effects.

The synergistic reason for the *in vivo* antidepressant effects of deDM and BUP may be that BUP inhibits deDM metabolizing enzymes, which in turn increases deDM exposure *in vivo*. Previous studies (Kotlyar et al., 2005) demonstrated that BUP is a primary CYP2D6 inhibitor, whereas DM is primarily metabolized by CYP2D6. Therefore, when BUP and DM are combined, BUP increases *in vivo* exposure to DM. As demonstrated in the mouse PK study presented in Table 3, BUP increased the exposure of DM *in vivo* in mice, further supporting the conclusion that BUP may improve *in vivo* stability by inhibiting the DM metabolizing enzyme CYP2D6. We have also performed similar PK studies in rats and dogs with similar results (Supplementary Tables S1, S2). At the same time, it can be seen that BUP also increased the exposure of deDM in mice, suggesting that BUP may also inhibit deDM metabolizing enzyme to improve *in vivo* stability, and deDM is presumably metabolized mainly by CYP2D6.

To further validate the synergistic effects of deDM and BUP *in vivo* in terms of pathomechanisms and to compare the differences with the DM + BUP combination, we used a reserpine-induced hypothermia model in rats and an ammonia-induced cough model in mice.

Patients with depression often experience dysregulation of body temperature. A rat model of reserpine-induced hypothermia can mimic this symptom and could help us determine whether antidepressant drugs can correct reserpine-induced decreases in body temperature. This animal model may also indirectly reflect the impact of the compounds on the alleviation of depressive symptoms. The FST and TST assess depression based on behavioral responses to inescapable stress conditions related to behavioral despair. In comparison, the reserpine-induced hypothermia model evaluates the antidepressant efficacy from a physiological perspective. It also involves changes in the endocrine system and in temperature regulation.

As shown in Figure 3, in the reserpine-induced hypothermia model in rats, deDM could dose-dependently elevate the body temperature of rats compared with the vehicle group, and deDM reached a significant elevation at the dose of 10 mg/kg. When deDM was combined with BUP, it further elevated the body temperature of rats, and the deDM + BUP (10 + 40 mg/kg) group had a significant elevation compared with the BUP group, which further suggests that deDM and BUP can synergistically enhance the antidepressant effect. Further comparative analysis showed that deDM + BUP (10 + 40 mg/kg) was superior to the combination of deDM + BUP (10 + 40 mg/kg) at increasing the body temperature of rats at the same dose. Based on the analysis of the PK results in mice in Table 3, this may be due to the higher stability of deDM than that of DM *in vivo*.

**FIGURE 5:**
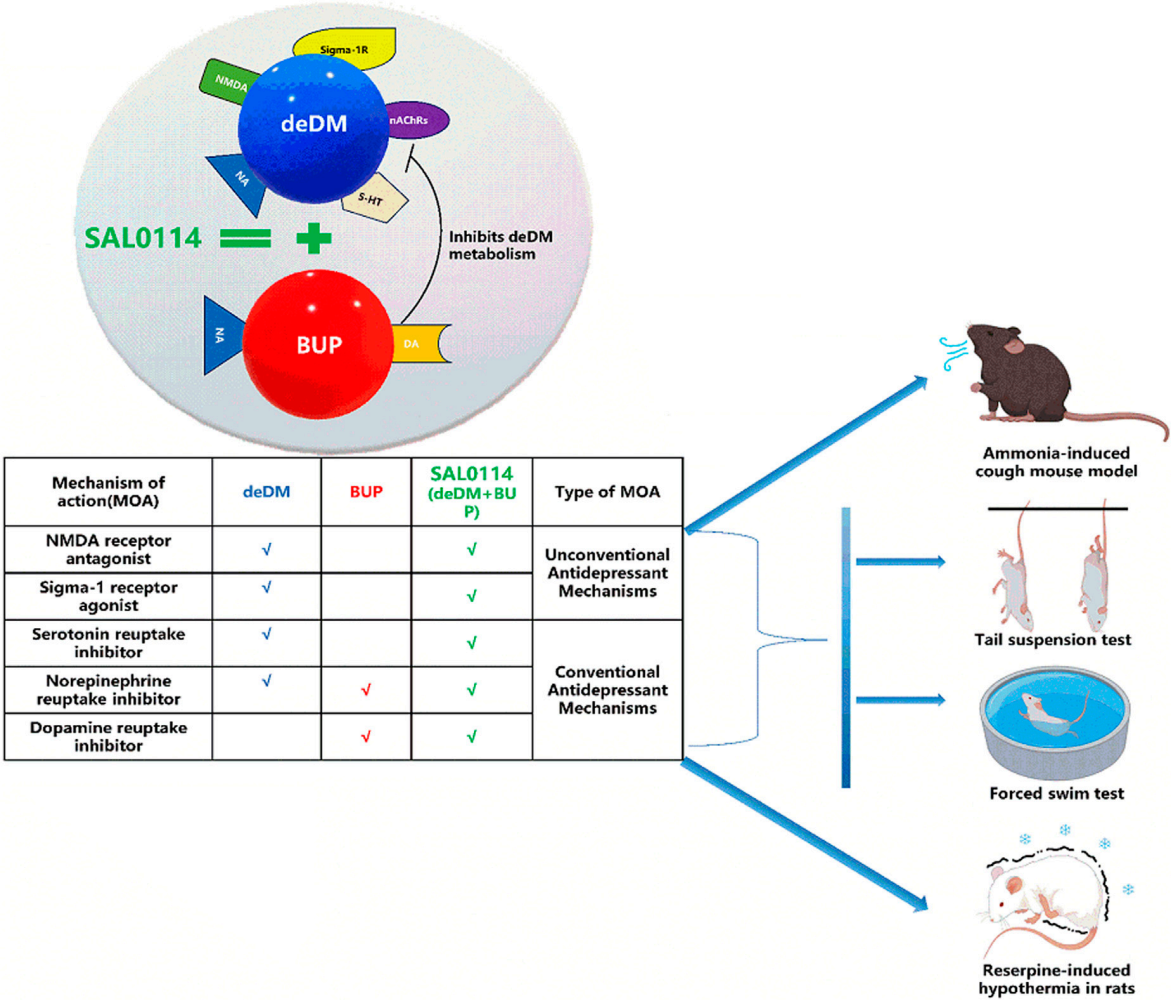
Graphical abstract.

In a reserpine-induced rat hypothermia model, reserpine is a vesicle uptake inhibitor. It can decrease neurotransmitter levels by inhibiting the reuptake of NA, DA, and 5-HT into vesicles, increasing the degradation of neurotransmitters by monoamine oxidase, and leading to symptoms of depression, such as hypothermia (Strawbridge et al., 2023). BUP is a transporter inhibitor of DA and NA. It can inhibit their reuptake into presynaptic neurons and increase the concentration of neurotransmitters in the synaptic gap (Bupropion, n. d.). In addition, as shown in Table 1, deDM and DM are both 5-HT and NA transporter inhibitors. It can inhibit the reabsorption of these neurotransmitters into presynaptic neurons and increase the concentration of neurotransmitters in the synaptic gap, thus achieving antidepressant effects and elevating the body temperature of rats. Moreover, as shown in Figure 5, there is complementarity in the targets of BUP and deDM. In other words, their effects on elevated neurotransmitter levels are complementary and may also contribute to the synergistic effect of antidepressants.

In the ammonia-induced cough mouse model, there was no significant difference in the antitussive effect of deDM compared to the vehicle group. However, the combination of deDM and BUP significantly suppressed coughing in mice in a dose-dependent manner. The antitussive effect of both doses of the deDM + BUP combination was greater than the arithmetic sum of the effects of BUP and deDM alone, further indicating the synergistic antitussive effect of deDM and BUP. In addition, the combination of deDM + BUP was more effective than the combination of DM + BUP in suppressing cough in mice at the same dose. As shown in Table 3, the *in vivo* PK study in mice could explain the synergistic effect of deDM and BUP, as well as the fact that the deDM + BUP combination had a better cough-suppressing effect than the DM + BUP combination. The *in vivo* PK study in mice showed that BUP increased the exposure of deDM, and the AUC0-last and Cmax of the deDM + BUP combination were higher than those of the DM + BUP combination at the same dose.

Previous studies (Brown et al., 2004; Canning, 2009) have shown that sigma-1R plays an important role in regulating the cough reflex. Therefore, this ammonia-induced cough mouse model can be used to reflect the agonistic activity of the compounds on sigma-1R. In addition to its involvement in the cough process, sigma-1R is involved in the pathogenesis of depression. Early investigations (Nguyen et al., 2014) have shown that sigma 1R agonists modulate neurotransmitter networks, signaling pathways, and brain activity related to the physiology of depression, and sigma 1R knockout animals display depression-like characteristics (Sałaciak and Pytka, 2022). Compared to typically prescribed antidepressant medications, Sigma 1R agonists may promote a quicker onset of antidepressant efficacy. This is supported (Ren et al., 2022) by the fact that sigma 1 receptor agonists, such as SA 4503, improved serotonergic neuronal activity in the dorsal spinal nucleus after just 2 days of treatment, whereas conventional antidepressants typically require at least 2 weeks of treatment. In addition, Sigma-1R regulates the synaptic transmission of NMDA receptors (Ryskamp et al., 2019). Together, these data suggest that sigma-1R can work independently and/or in conjunction with other pathways (e.g., monoaminergic systems) to produce more rapid antidepressant effects and that DM or deDM, by exploiting these mechanisms, may produce faster effects than conventional antidepressants in depressed patients.

In conclusion, our *in vitro* and *in vivo* data collectively demonstrate that deuterium substitution does not alter DM activity against antidepressant targets of action but enhances the metabolic stability of DM both *in vitro* and *in vivo*. BUP may synergistically enhance antidepressant effects by inhibiting the metabolizing enzymes of deDM, thereby further increasing deDM exposure *in vivo*. In addition, there are complementary antidepressant targets of BUP and deDM that may contribute to the synergistic antidepressant effects. Importantly, the combination of deDM and BUP offers more than just treatment for depression. They treat depression by boosting traditional monoamine neurotransmitters, such as 5-HT, NE, and DA, as well as by acting on sigma-1 and NMDA receptors. This novel mode of action can achieve rapid antidepressant effects in the clinical setting.

The deDM + BUP combination had a superior antidepressant effect compared to the DM + BUP combination at equivalent doses. This is attributed to the higher exposure to deDM in the deDM + BUP combination relative to DM in the DM + BUP combination. Furthermore, because deDM has higher stability *in vitro* and *in vivo* than DM, it has a lower potential for metabolic metabolites, which are thought to have negative effects on clinical nerves. These results imply that deDM + BUP may possess a better efficacy and safety profile than DM + BUP. The deDM + BUP combination is currently undergoing multiple phase 1/2 clinical trials in China (CTR20220758, CTR20222884, and CTR20240090). In these clinical trials, the deDM + BUP combination demonstrated favorable safety and tolerability profiles. We believe that it can provide safer and more effective treatment for patients with depression.

## Data Availability

The raw data supporting the conclusions of this article will be made available by the authors, without undue reservation.

## Supplementary material

SUPPLEMENTARY FIGURE S1 DM, deDM, DX, and deDX binding effects on NMDA receptors. **(A)** dextromethorphan (DM). **(B)** deuterated dextromethorphan (deDM). **(C)** dextrorphan (DX). **(D)** deuterated dextrorphan (deDX).

SUPPLEMENTARY FIGURE S2 Binding effects of DM, deDM, DX, and deDX on the Sigma-1 receptor. **(A)** dextromethorphan (DM). **(B)** deuterated dextromethorphan (deDM). **(C)** dextrorphan (DX). **(D)** deuterated dextrorphan (deDX).

SUPPLEMENTARY FIGURE S3 Binding effects of DM, deDM, DX, and deDX on α3β4 nicotinic acetylcholine receptors. **(A)** dextromethorphan (DM). **(B)** deuterated dextromethorphan (deDM). **(C)** dextrorphan (DX). **(D)** deuterated dextrorphan (deDX).

SUPPLEMENTARY FIGURE S4 Binding effects of DM, deDM, DX, and deDX on serotonin transporter (SERT). **(A)** dextromethorphan (DM). **(B)** deuterated dextromethorphan (deDM). **(C)** dextrorphan (DX). **(D)** deuterated dextrorphan (deDX).

SUPPLEMENTARY FIGURE S5 Binding effects of DM, deDM, DX, and deDX on norepinephrine transporter (NET). **(A)** dextromethorphan (DM). **(B)** deuterated dextromethorphan (deDM). **(C)** dextrorphan (DX). **(D)** deuterated dextrorphan (deDX).
