# Supplementary material for: SAL0114: a novel deuterated dextromethorphan-bupropion combination with improved antidepressant efficacy and safety profile
Source: Front Pharmacol. 2024 Sep 24;15:1464564. doi: 10.3389/fphar.2024.1464564 (PMC11462627; doi:10.3389/fphar.2024.1464564)
Supplement: Supplementary file 1 [file Table1.DOCX]

**Supplementary Table 1** Pharmacokinetic parameters of DM or deDM in male SD rat after oral administratotion of the following different gourps of treatment

| Group | Dose (mg/kg) | AUC_0-last_ (h*ng/ml) | C_max_ (ng/ml) |
| --- | --- | --- | --- |
| DM | 50 | 194 | 56 |
| deDM | 50 | 374 | 84 |
| deDM+BUP | 50+300 | 1370 | 226 |

Results expressed as mean, n=3 per group
