# Supplementary figures and images for: SAL0114: a novel deuterated dextromethorphan-bupropion combination with improved antidepressant efficacy and safety profile
Source: Front Pharmacol. 2024 Sep 24;15:1464564. doi: 10.3389/fphar.2024.1464564 (PMC11462627; doi:10.3389/fphar.2024.1464564)

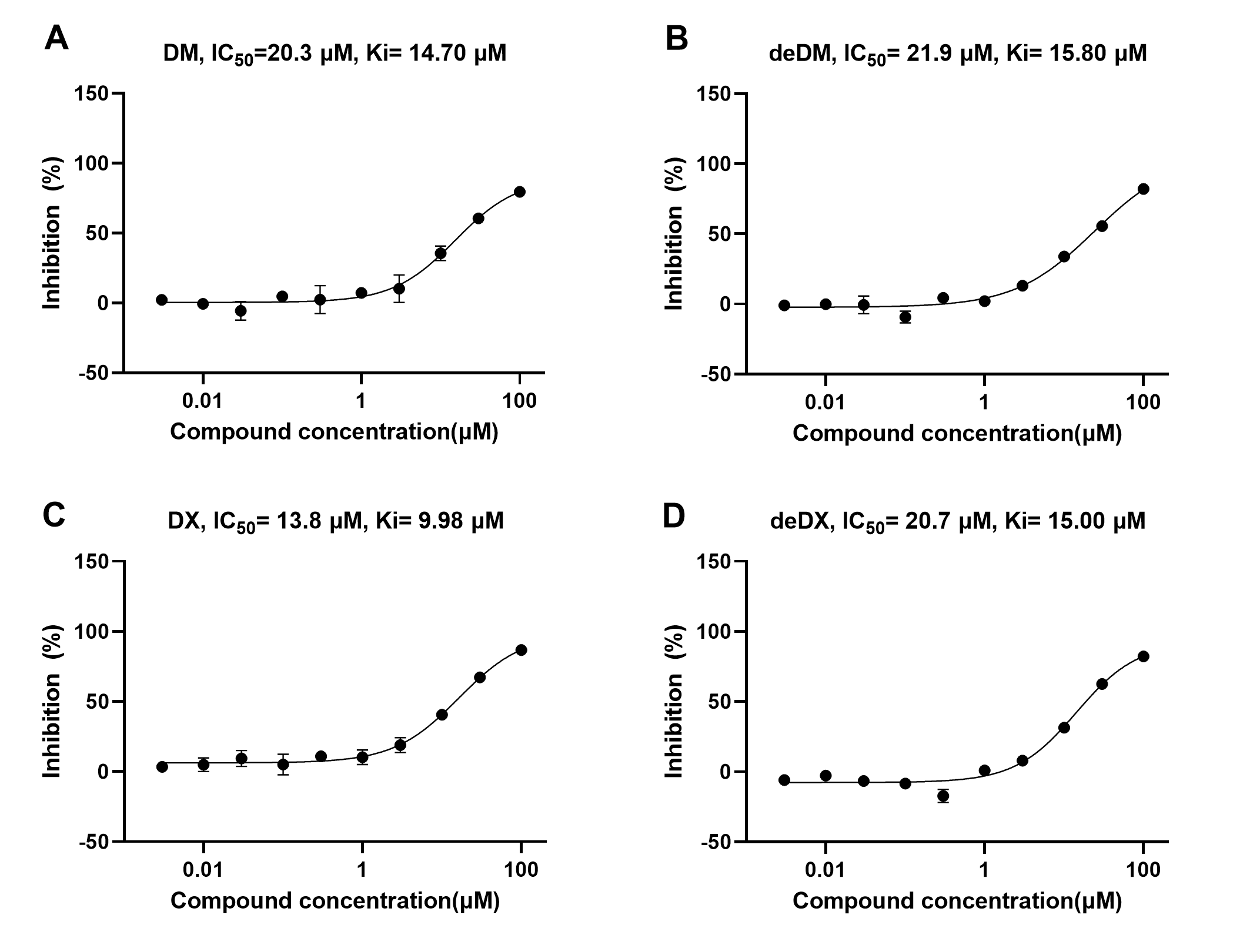

Supplement: Supplementary file 2 [file Image3.tif]

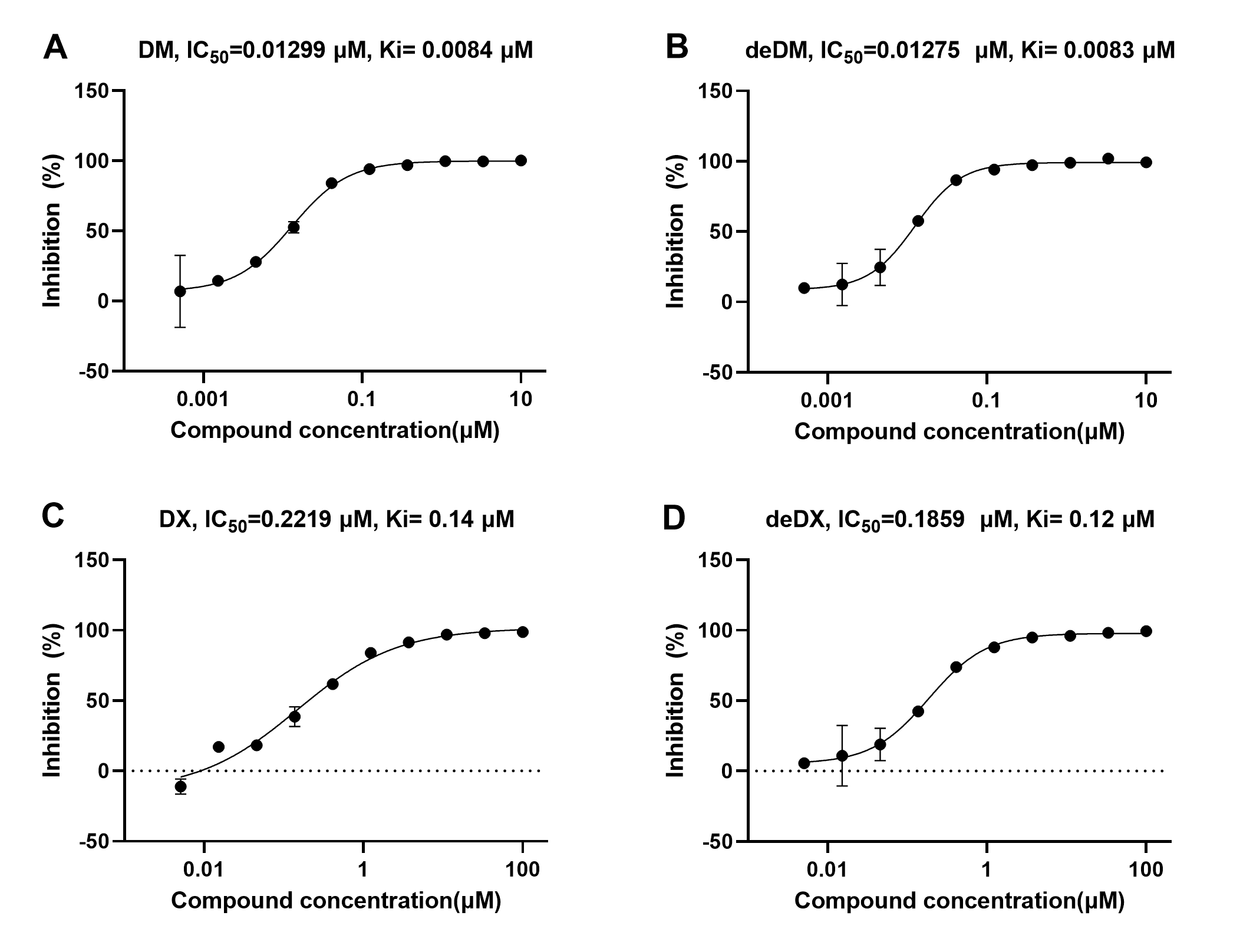

Supplement: Supplementary file 3 [file Image4.tif]

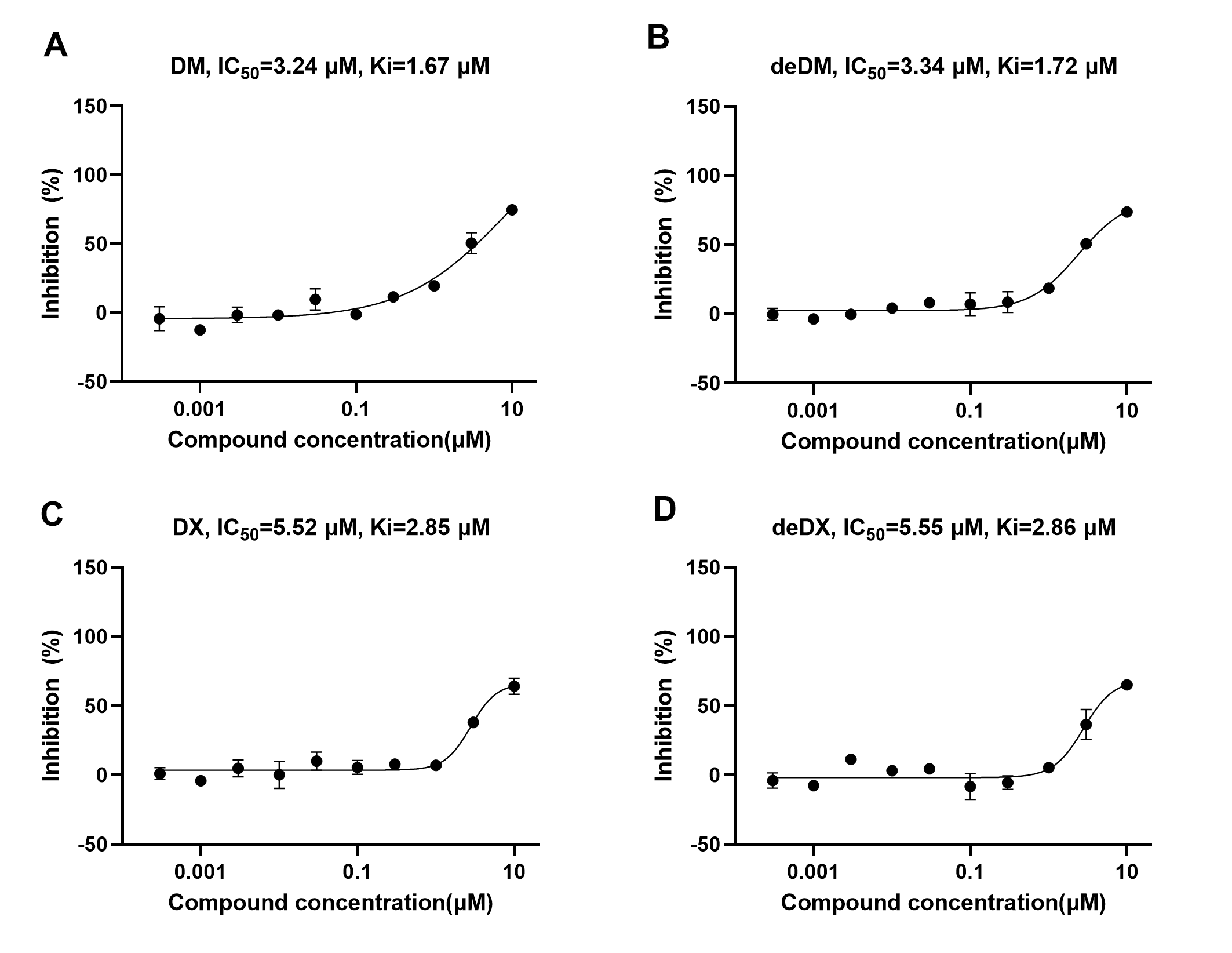

Supplement: Supplementary file 4 [file Image2.tif]

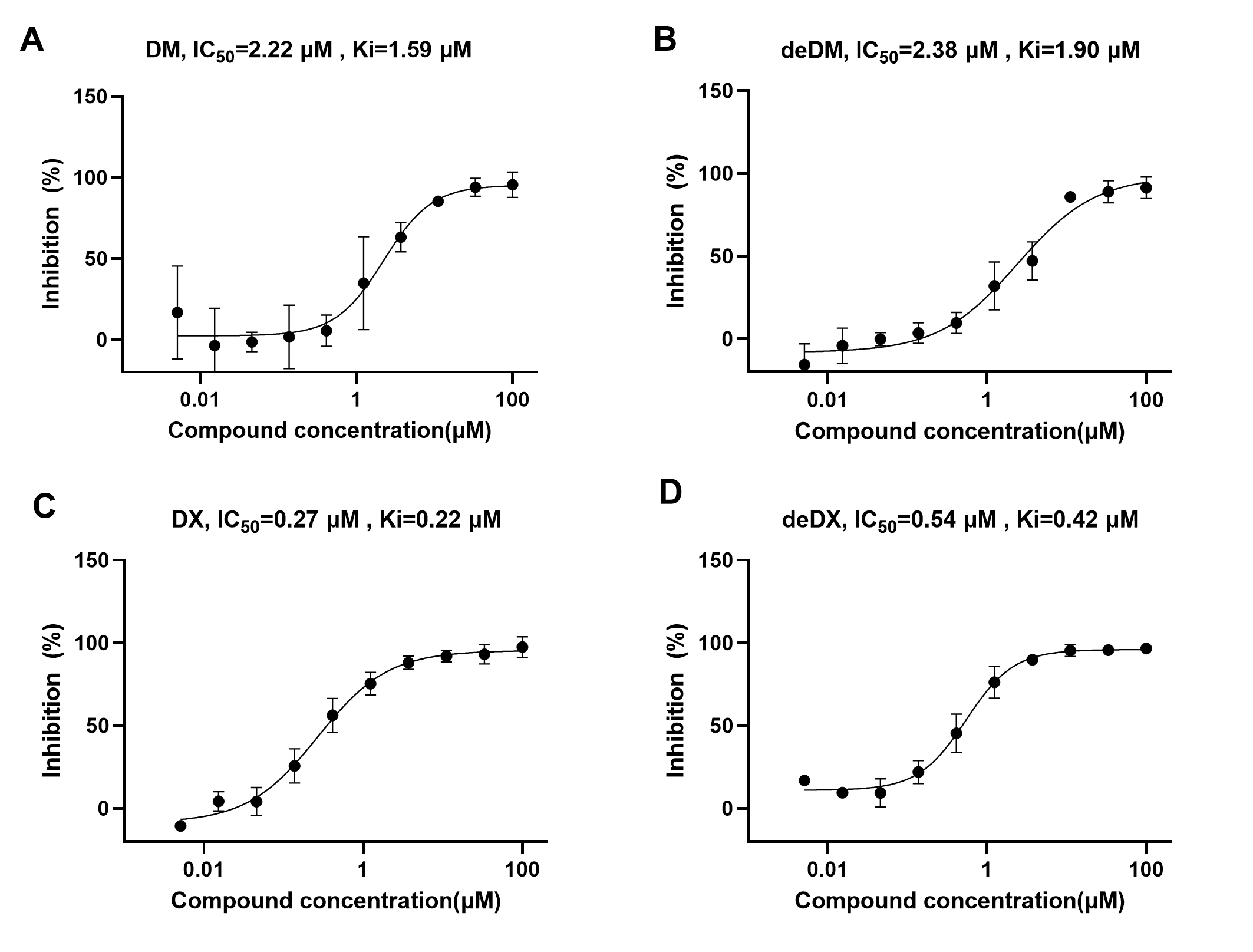

Supplement: Supplementary file 5 [file Image1.tif]

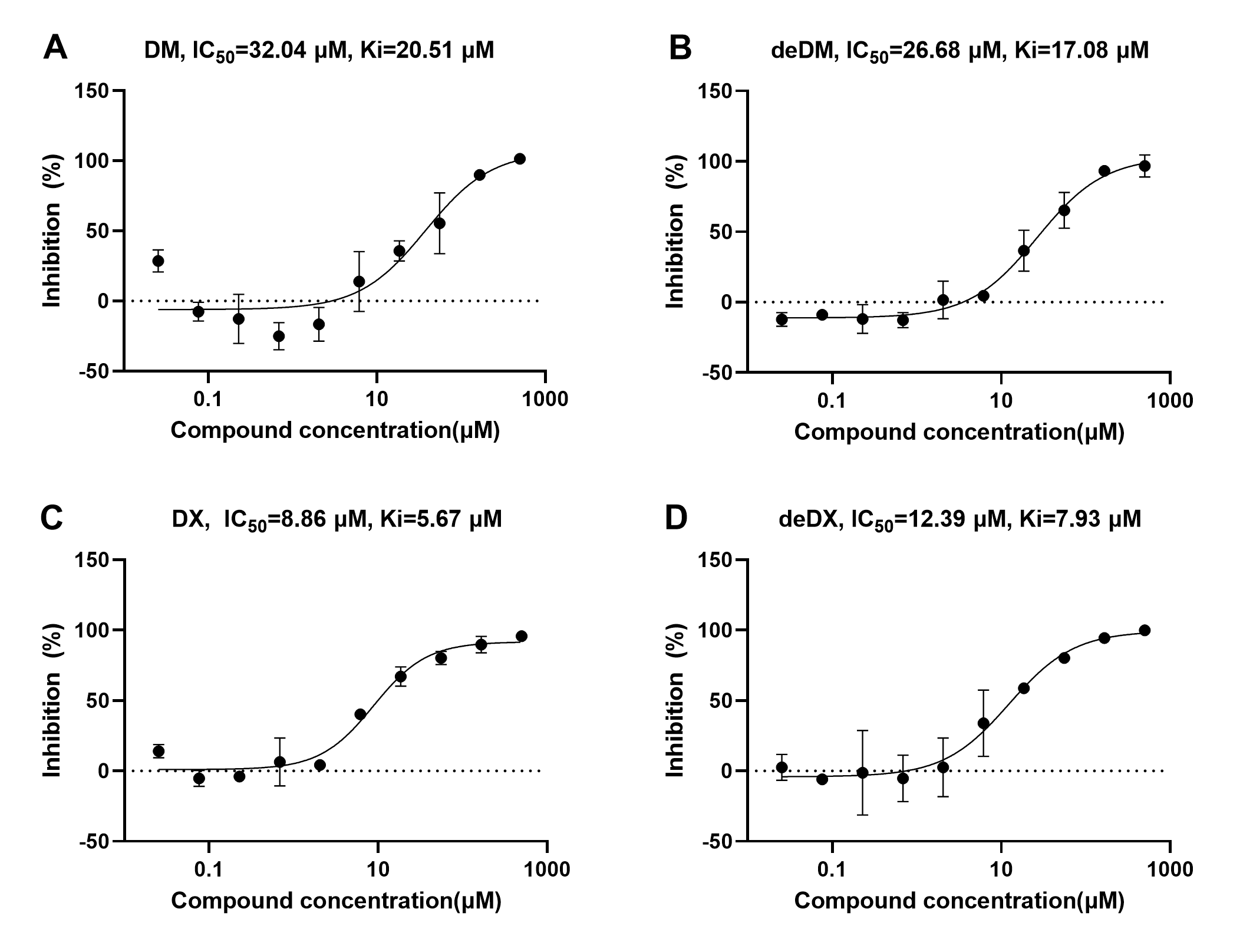

Supplement: Supplementary file 7 [file Image5.tif]
