# Supplementary material for: SAL0114: a novel deuterated dextromethorphan-bupropion combination with improved antidepressant efficacy and safety profile
Source: Front Pharmacol. 2024 Sep 24;15:1464564. doi: 10.3389/fphar.2024.1464564 (PMC11462627; doi:10.3389/fphar.2024.1464564)
Supplement: Supplementary file 6 [file Table2.DOCX]

**Supplementary Table 2** Pharmacokinetic parameters of DM or deDM in beagle dog after oral administratotion of the following different gourps of treatment

| Group | Dose (mg/kg) | AUC_0-last_ (h*ng/ml) | C_max_ (ng/ml) |
| --- | --- | --- | --- |
| deDM | 14 | 383(♂); 153(♀) | 157(♂); 60(♀) |
| deDM+BUP | 14+58 | 935(♂); 622(♀) | 250(♂); 216(♀) |
| DM+BUP | 14+58 | 618(♂); 454(♀) | 166(♂); 138(♀) |

Results expressed as mean, n=3 per group
